# Conveyance Contact Investigation for Imported Middle East Respiratory Syndrome Cases, United States, May 2014

**DOI:** 10.3201/eid2309.170365

**Published:** 2017-09

**Authors:** Susan A. Lippold, Tina Objio, Laura Vonnahme, Faith Washburn, Nicole J. Cohen, Tai-Ho Chen, Paul J. Edelson, Reena Gulati, Christa Hale, Jennifer Harcourt, Lia Haynes, Amy Jewett, Robynne Jungerman, Katrin S. Kohl, Congrong Miao, Nicolette Pesik, Joanna J. Regan, Efrosini Roland, Chris Schembri, Eileen Schneider, Azaibi Tamin, Kathleen Tatti, Francisco Alvarado-Ramy

**Affiliations:** Centers for Disease Control and Prevention, Atlanta, Georgia, USA

**Keywords:** Aircraft, bus, commercial travel, contact tracing, coronavirus, disease notification, disease outbreaks, viruses, Middle East respiratory syndrome coronavirus, MERS-CoV, travel, travelers, United States, Saudi Arabia, United Kingdom, respiratory infections, zoonoses

## Abstract

In 2014, the Centers for Disease Control and Prevention conducted conveyance contact investigations for 2 Middle East respiratory syndrome cases imported into the United States, comprising all passengers and crew on 4 international and domestic flights and 1 bus. Of 655 contacts, 78% were interviewed; 33% had serologic testing. No secondary cases were identified.

Two cases of imported Middle East respiratory syndrome (MERS) in the United States were confirmed in May 2014 in travelers from Saudi Arabia (*1*). Both persons were symptomatic at the time of travel. The Centers for Disease Control and Prevention (CDC) conducted large-scale (entire plane) contact investigations for the affected flights and for an interstate bus.

## The Study

The investigation had 3 objectives: 1) notify travelers, 2) identify symptomatic contacts and facilitate prompt evaluation and isolation, and 3) determine the extent of onboard transmission. CDC approved the protocol as nonresearch.

We obtained passenger information and distributed it to state health departments as described (*2*). Foreign public health authorities were notified for US citizens abroad and foreign passport holders located outside the United States within the 14-day incubation period.

State health departments, CDC, or airline occupational health staff interviewed contacts using a standard questionnaire. Contacts interviewed within 14 days after exposure were advised to monitor themselves for fever and respiratory symptoms and to report symptoms to their state or local health department. Symptomatic contacts were reinterviewed about coexisting conditions and other exposure risks. When clinically indicated, state health departments coordinated specimen collection for testing by real-time reverse transcription PCR (rRT-PCR) (Table 1).

**Table 1:** CDC case and contact definitions during MERS contact investigations*

Status	Definition	Signs and symptoms
Index case	Laboratory-confirmed MERS-CoV infection in a person who traveled by commercial aircraft or bus while symptomatic. A traveler who tested positive for MERS-CoV was considered to have been contagious if symptomatic while on the conveyance.	Fever (>38°C [>100.4°F]) or feverishness;† or symptoms of acute respiratory illness (i.e., cough, shortness of breath, rhinorrhea, sore throat); or myalgia, malaise; or gastrointestinal symptoms (i.e., nausea, diarrhea, or vomiting).
Conveyance contact	A person who traveled on the same conveyance as the index case-patient and who had:	
Category 1	Compatible symptoms within 2–14 d after the flight or bus ride.	Fever (>38°C [100.4°F]) or feverishness; or symptoms of acute respiratory illness (i.e., cough, shortness of breath, rhinorrhea, sore throat); or myalgia, malaise; or gastrointestinal symptoms (i.e., nausea, diarrhea, or vomiting).
Category 2	Unrelated symptoms.	An illness before the flight or an illness with onset either <2 d or >14 d after the flight; or symptoms attributable to a chronic illness; or symptoms that were musculoskeletal, neurologic, or dermatologic in origin.
Category 3	No symptoms.	
Patient under investigation‡	Clinical features (severe or milder illness), including fever and respiratory symptoms, and epidemiologic risk factors, including travel, healthcare setting, and contact history.	
Incubation period for MERS-CoV infection	2–14 d after exposure.	

*CDC, Centers for Disease Control and Prevention; MERS, Middle East respiratory syndrome; MERS-CoV, MERS coronavirus. †A subjective sense of fever. ‡From (*3*).

With contacts’ consent, serum specimens were collected at least 14 days after exposure. Serologic tests were a recombinant MERS coronavirus (MERS-CoV) nucleocapsid protein–based ELISA, followed by confirmatory testing for MERS-CoV–specific antibodies by immunofluorescence assay and microneutralization test on ELISA-positive serum specimens. Serologic assays were developed and performed at CDC, and microneutralization testing was done in a BioSafety Level 3 laboratory at CDC. Symptomatic contacts (contact definition category 1) were tested at state health department laboratories using the CDC MERS-CoV rRT-PCR (*4*).

Index case-patient 1 was a 65-year-old resident of Saudi Arabia who developed myalgia, fatigue, and a low-grade fever around April 18. On April 24, he flew from Riyadh to London, UK (Boeing 747–400, flight time 6 h 50 min), then London to Chicago, IL, USA (Boeing 767–300, 8 h 45 min). He then traveled to Indiana by bus (1 h 10 min). On April 27, cough, shortness of breath, increasing fever, and rhinorrhea developed; he was hospitalized April 28. MERS was diagnosed May 1 by the Indiana State Department of Health and confirmed May 2 by CDC (*1*).

Index case-patient 2, unconnected to case-patient 1, was a 43-year-old resident of Saudi Arabia who traveled on 2 international and 2 domestic US flights on May 1. He felt ill on his Riyadh–London flight (Boeing 777–300ER, 6 h 30 min); fever, chills, and myalgia developed on a flight from London to Boston, MA, USA (Boeing 767–400, 7 h 40 min) and cough on the domestic flights: Boston–Atlanta, GA, USA (McDonnell Douglas D-90, 2 h 50 min) and Atlanta–Orlando, FL, USA (Boeing 757, 1 h 30 min). On May 9, he sought care at a Florida emergency department with fever, cough, chills, and myalgia. Bilateral crackles were noted on exam; chest radiograph was normal. On May 11, the Florida Department of Health diagnosed MERS, subsequently confirmed by CDC (*1*).

**Figure 1:**
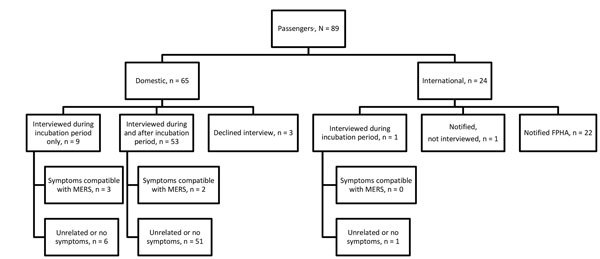
Flowchart of aircraft passengers exposed to index case-patient 1 in investigation of 2 imported US cases of Middle East respiratory syndrome, by location at time of notification, May 2014. Of all passengers, 78 (88%) were on the London–Chicago flight, 9 (10%) on the Riyadh–London flight, and 2 (2%) on the Riyadh–London and London–Chicago flights. Domestic passengers were assigned to state health departments for follow-up if contact information indicated they lived in that state; CDC assumed responsibility for interviewing passengers if they lacked contact information that would enable state health department assignment. One US citizen was interviewed by CDC while traveling abroad; 1 US citizen with dual citizenship on the Riyadh–London flight was notified by CDC but already had been interviewed by authorities in the country of residence. FPHA notifications were made for foreign passport holders and US citizens living or traveling abroad. The incubation period for MERS is 2–14 days after exposure. Symptoms compatible with MERS were fever (>38°C [>100.4°F]), feverishness, symptoms of acute respiratory illness (i.e., cough, shortness of breath, rhinorrhea, sore throat), myalgia, malaise or gastrointestinal symptoms 2–14 days after travel on the same conveyance as the index case-patient. CDC, Centers for Disease Control and Prevention; FPHA, foreign public health authority; MERS, Middle East respiratory syndrome; MERS-CoV, MERS coronavirus.

**Figure 2:**
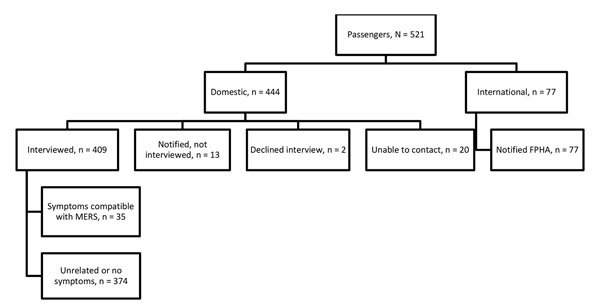
Flowchart of aircraft passengers exposed to index case-patient 2 in investigation of 2 imported US cases of Middle East respiratory syndrome, by location at time of notification, May 2014. Of all passengers 188 (36%) were on the London–Boston flight, 158 (30%) on the Boston–Atlanta flight, and 175 (34%) on the Atlanta–Orlando flight. Domestic passengers were assigned to state health departments for follow-up if contact information indicated they lived in that state. CDC assumed responsibility for interviewing passengers if they lacked contact information that would enable state health department assignment. A total of 337 contacts were interviewed late in the incubation period (days 11–14 after exposure) and were not contacted for a follow-up interview after the incubation period; remaining contacts were interviewed after the incubation period. FPHA were notified for foreign passport holders and US citizens living or traveling abroad. The incubation period for MERS is 2–14 days after exposure. Symptoms compatible with MERS were fever (>38°C [100.4°F]), feverishness, symptoms of acute respiratory illness (i.e., cough, shortness of breath, rhinorrhea, sore throat), myalgia, malaise, or gastrointestinal symptoms in persons who traveled on the same conveyance as the index case-patient. CDC, Centers for Disease Control and Prevention; FPHA, foreign public health authority; MERS, Middle East respiratory syndrome.

CDC investigated the 2 international flights inbound to the United States and 2 domestic flights. Nine additional US contacts were provided for the Riyadh–London flight of case-patient 1 and none for case-patient 2. CDC identified a total of 655 contacts for both persons. For case-patient 1, these were 89 passengers (Figure 1) and 12 crew members. The bus company reported 10 potential contacts but was able to identify only the driver and 5 passengers who had paid by credit card. For case-patient 2, a total of 521 passengers and 23 crew members were identified for the flights investigated by CDC (Figure 2).

For case-patient 1, passenger-locating information was provided to 17 health departments (1–12/state) the day MERS was confirmed, 8 days after exposure; interviews were sought during and after the incubation period. CDC notified 1 country for 22 passengers. For case-patient 2, contact information provided to 35 health departments (1–80/state), 11–12 days after the flight; 1 interview was sought. CDC notified 15 countries for 77 passengers.

Of the total 655 contacts, 631 (96%) were notified, of whom 512 (81%) were interviewed an average of 2.8 days after MERS confirmation. Of these, 435 (85%) reported no symptoms, 42 (8%) MERS-compatible symptoms, and 35 (7%) unrelated symptoms (Table 2).

**Table 2:** Characteristics of passenger and crew contacts during 2 MERS conveyance contact investigations, May 2014*

Contact characteristic	Case-patient 1					Case-patient 2†					Total
	RUH–LHR,‡ LHR–ORD	Crew§	Bus¶	Total		LHR–BOS	BOS–ATL	ATL–MCO	Crew§	Total	
No. contacts	89	12	10	111		188	158	175	23	544	655
Level of contact, no. (%)											
** Consented to interview#**	63 (71)	12 (100)	5 (50)	80 (72)		134 (71)	136 (86)	139 (79)	23 (100)	432 (79)	512 (78)
Notified, not interviewed**	1†† (1)	NA	NA	1 (0.9)		2 (1)	9 (6)	2 (1)	NA	13 (2)	14 (2)
** FPHA notified**	22 (25)	NA	NA	22 (20)		46 (24)	3 (2)	28 (16)		77 (14)	99 (15)
** Declined interview**	3 (3)	NA	NA	4 (3.6)		2 (1)	NA	NA	NA	2 (0.4)	6 (0.9)
** Unable to contact**‡‡	NA	NA	NA	4 (3.6)		4 (2)	10 (6)	6 (3)	NA	20 (4)	24 (4)
Age**											
** Mean, y**	44.1	NA	55.5	44.8		47.8	41.1	35.9	NA	41	41.5
** Median, y**	47	NA	56.5	48		47	41	37	NA	42	43
** Range (Q1**–Q3), y	35–55	NA	40.5–70.5	32–56		36–55	31.5–50.5	21–49.5	NA	30–53	30–53
No. unknown	3	12	1	16		9	4	15	23	50	66
Sex, no. (%)**											
** M**	35 (56)	4 (33)	4 (80)	43 (54)		89 (66)	94 (69)	68 (49)	NA	251 (58)	294 (57)
** F**	28 (44)	5 (42)	1 (20)	34 (43)		43 (33)	41 (30)	71 (51)	1 (4)	156 (36)	190 (37)
** Unknown**	NA	3 (25)	NA	3 (4)		2 (1)	1 (0.7)	NA	22 (96)	25 (6)	28 (5)
Passengers who changed seats, no. (%)§§,¶¶	2 (3)	NA	NA	2 (3)		1 (0.7)	NA	NA	NA	1 (0.2)	3 (0.6)
Consent for serologic testing, no. (%)¶¶											
** Yes**	NA	NA	4 (80)	45 (56)		91 (68)	116 (85)	101 (73)	NA	308 (71)	353 (69)
** No**	NA	NA	NA	10 (13)		34 (25)	16 (12)	28 (20)	23 (100)	78 (18)	88 (17)
** Unknown**	12 (100)	12 (100)	1 (20)	25 (31)		9 (7)	4 (3)	10 (7)	NA	46 (11)	71 (14)

*FPHA, foreign public health authorities; LHR, London Heathrow Airport (London, UK); BOS, Boston Logan International Airport (Boston, MA, USA); ATL, Hartsfield-Jackson Atlanta International Airport (Atlanta, GA, USA); MCO, Orlando International Airport (Orlando, Florida, USA); NA, not applicable; ORD, Chicago O'Hare International Airport (Chicago, IL, USA); Q, quartile; RUH, King Khalid International Airport (Riyadh, Saudi Arabia). †CDC was not contacted about any US citizens exposed to case-patient 2 on the Riyadh–London flight. ‡Includes 9 US citizens who were passengers on RUH–LHR flight only and 2 US citizens who were passengers on both RUH–LHR and LHR–ORD flights. §Includes all cabin crew and pilots for all flights. ¶Includes bus driver. #New denominator used for calculating all passenger demographic information. **Defined as either a notification sent to FPHA for a contact who was not interviewed while in the United States, certified mail sent to a valid address, voicemail left for a working phone number, or email sent to a valid email address. ††One US citizen with dual citizenship on Riyadh–London flight was notified by CDC but had already been interviewed by authorities in country of residence. ‡‡Unable to locate or contact because of lack of or incorrect contact information. §§Seat was reassigned if passenger sat in new seat for more than half of flight and could clearly articulate the new seat number or location. ¶¶Denominator includes only contacts who consented to interview.

Of 42 contacts with MERS-compatible symptoms, 7 reported acute respiratory illness with fever/feverishness, 32 acute respiratory illness without fever, 4 myalgia, 4 gastrointestinal symptoms, and 2 malaise. For 12 (29%), rRT-PCR was performed; MERS-CoV RNA was not detected. One contact who tested negative had been in the Middle East during the 14 days before the flight but reported no exposures of concern.

Blood was drawn for serum testing a mean of 33 days after exposure (median 21 days, range 9–90 days) from 218 (62%) aircraft passengers; 11 had unknown collection dates. Twelve (5%) specimens were collected within 14 days, 11 on day 12 or 13; the remainder were collected 14–90 days after the flight. Serologic test results were available for 11 (25%) passengers from both flights who sat within 2 rows of the case-patient and for 13 passengers who reported MERS-compatible symptoms. All serum tested negative for antibodies to MERS-CoV.

All flight crew were interviewed and reported no or unrelated symptoms. The bus driver and 4 of 5 passengers were interviewed and remained asymptomatic. No flight crew or bus contacts provided serum.

## Conclusions

Close collaboration between state and local health departments, CDC, airline and bus industries, and federal partners was critical to rapidly complete these resource-intensive investigations. The 3 protocol objectives were met: achieving a high rate of timely notifications, identifying and evaluating symptomatic contacts, and using serology to detect transmission. The investigation detected no transmission on any of the conveyances. Concurrent household and healthcare facility contact investigations for these cases also did not identify MERS-CoV transmission (*5*).

At least 8 other aircraft and 2 bus investigations have been reported, totaling >400 evaluated contacts (*6**–**13*). Index case-patient symptoms have varied; flight times ranged from 1.5 to 10 hours. Contact definitions ranged from 2 adjacent seats in all directions to the entire plane; most common was within 2 rows of the index case-patient. Several investigations included laboratory testing of symptomatic or asymptomatic contacts. No transmission on aircraft or buses has been documented.

Limitations of our investigation included incomplete follow-up, self-reported symptoms, and potential recall bias. Cases may have been missed because a small number of travelers were interviewed or had specimens collected only during the 14-day incubation period.

The results of this and other investigations suggest the risk for MERS-CoV transmission on conveyances is low. Recent publications concluded that aircraft contact tracing required extensive preparation, resources, and passenger compliance; was an inconvenience to passengers; had mixed outcomes (*14*); and caused psychological distress to contacts (*12*). Our investigation required substantial resources of airlines, a bus company, local and state health departments, federal agencies, and foreign public health authorities. For future aircraft contact investigations for MERS, CDC will include only passengers seated within 2 rows of the index case-patient, although modifications may be made depending on circumstances. Comprehensive conveyance contact investigations with laboratory evaluation can guide future public health practice for emerging communicable diseases.
